# Effect of Gestational Diabetes Mellitus History on Future Pregnancy Behaviors: The Mutaba’ah Study

**DOI:** 10.3390/ijerph18010058

**Published:** 2020-12-23

**Authors:** Nasloon Ali, Aysha S. Aldhaheri, Hessa H. Alneyadi, Maha H. Alazeezi, Sara S. Al Dhaheri, Tom Loney, Luai A. Ahmed

**Affiliations:** 1 Institute of Public Health, College of Medicine and Health Sciences, United Arab Emirates University, P.O. Box 17666, Al Ain, UAE; nasloona@uaeu.ac.ae (N.A.); 201505948@uaeu.ac.ae (A.S.A.); 201501873@uaeu.ac.ae (H.H.A.); 201501621@uaeu.ac.ae (M.H.A.); 201504033@uaeu.ac.ae (S.S.A.D.); 2 College of Medicine, Mohammed Bin Rashid University of Medicine and Health Sciences, P.O. Box 505055, Dubai, UAE; tom.loney@mbru.ac.ae; 3 Zayed Centre for Health Sciences, United Arab Emirates University, P.O. Box 17666, Al Ain, UAE

**Keywords:** gestational diabetes mellitus, maternal health, prenatal care, pregnancy, United Arab Emirates

## Abstract

Gestational diabetes mellitus (GDM) increases the risk of adverse pregnancy outcomes in any pregnancy and recurrence rates are high in future pregnancies. This study aims to investigate the effect of self-reported history of previous GDM on behaviors in a future pregnancy. This is an interim cross-sectional analysis of the pregnant women who participated in the Mutaba’ah Study between May 2017 and March 2020 in the United Arab Emirates. Participants completed a baseline self-administered questionnaire on sociodemographic and pregnancy-related information about the current pregnancy and previous pregnancies. Regression models assessed the relationships between self-reported history of GDM and pre-pregnancy and pregnancy behaviors in the current pregnancy. Out of 5738 pregnant parous women included in this analysis, nearly 30% (*n* = 1684) reported a history of GDM in a previous pregnancy. Women with a history of previous GDM were less likely to plan their current pregnancies (adjusted odds ratio (aOR): 0.84, 95% confidence interval (CI) 0.74–0.96) and more likely to be worried about childbirth (aOR: 1.18, 95% CI 1.03–1.36). They had shorter interpregnancy intervals between their previous child and current pregnancy (aOR: 0.88, 95% CI 0.82–0.94, per SD increase). There were no significant differences between women with and without a history of GDM in supplement use, sedentary behavior, or physical activity before and during this current pregnancy. Nearly a third of parous pregnant women in this population had a history of GDM in a previous pregnancy. Pregnant women with a previous history of GDM were similar to their counterparts with no history of GDM in the adopted pre-pregnancy and prenatal health behaviors. More intensive and long-term lifestyle counseling, possibly supported by e-health and social media materials, might be required to empower pregnant women with a history of GDM. This may assist in adopting and maintaining healthy prenatal behaviors early during the pregnancy or the preconception phase to minimize the risk of GDM recurrence and the consequential adverse maternal and infant health outcomes.

## 1. Introduction

Gestational diabetes mellitus (GDM) is one of the most common conditions during pregnancy affecting approximately one in six live births globally [1] based on different diagnostic criteria and population characteristics. A diagnosis of GDM can lead to several unwanted adverse outcomes in both the mother and the child. Pregnant women are at an increased risk of pre-eclampsia or eclampsia, cesarean section, preterm deliveries, induction of birth, longer hospital stay after delivery, and lower quality of life [2,3,4,5,6,7]. Children of GDM mothers are often born heavier than their peers, have increased risk of complications at delivery such as shoulder dystocia and hyperglycemia, and typically experience weight management issues during childhood [2,6,8,9,10,11]. Both the mother and child are at an increased risk of developing type II diabetes mellitus in the future [12,13]. Nevertheless, there are many behaviors and interventions that can prevent these adverse outcomes and increase the likelihood of women with GDM having healthy pregnancies and deliveries, in addition to their children leading a healthy and uncomplicated childhood [1,14].

During their antenatal care (ANC) appointments and throughout their pregnancies, women diagnosed with GDM are often provided with lifestyle counseling on how to improve their health as an adjunct to prescribed medication. This may include lifestyle changes such as dietary restrictions on highly processed, carbohydrate-dense foods as well as food and beverages with a high glycemic index [15,16]. In addition, advice on optimal physical activity before and during their pregnancy [17] and the consumption of specific supplements that can prevent any nutrient deficiencies that have been linked to unfavorable health outcomes in the child [18,19,20] may be provided. Women are also advised to continue a healthy postpartum lifestyle to prevent the development of GDM in future pregnancies. This is especially important as approximately 50–73% of women with GDM develop GDM in a future pregnancy [21,22,23]. Risk factors and outcomes associated with GDM have been widely investigated. However, there is a lack of evidence on how women previously diagnosed with GDM behave during the rest of their reproductive careers.

In the United Arab Emirates (UAE), comprehensive guidelines have been implemented by health authorities to ensure safe pregnancies and deliveries. Women are regularly monitored, provided hemoglobin A1C checks, and are provided glucometers if diagnosed with GDM and required to monitor their glucose levels. Pregnant women are also provided with lifestyle counseling to improve diet and physical activity and manage weight status throughout the pregnancy [24]. However, there is a lack of evidence on the long-term implementation of these healthy behaviors by women with a history of previous GDM during their future pregnancies. A recent systematic review of maternal and birth cohort studies in the Gulf Cooperation Council countries, including the UAE, found approximately 20 publications focusing on GDM [25]. However, there were no studies exploring the influence of GDM history on health behaviors during future pregnancies. Hence, the aim of the study was to investigate the influence of a history of GDM on the pre-pregnancy and prenatal behaviors of women during their current pregnancy in a prospective mother and child cohort study in the UAE.

## 2. Materials and Methods

This interim cross-sectional analysis is based on the pregnant women who participated in the Mutaba’ah Study, which is an ongoing prospective mother and child cohort study in Al Ain city, UAE. The overarching aim of the Mutaba’ah (which means to “follow-up” in Arabic) Study is to systematically recruit 10,000 pregnant women from the Emirati population during their ANC visits at the three major health institutions in the city. All pregnant women from the Emirati population, who are aged 18 years and above, resident in Al Ain, and able to provide informed consent, and their newborns are eligible to participate in the study. The Mutaba’ah Study has been described in detail elsewhere [26]. The study was approved by the research ethics committees of the United Arab Emirates University (ERH-2017-5512), Al Ain Hospital (AAHEC-03-17-058), and Tawam Hospital (IRR–494) and was in complete agreement with the Declaration of Helsinki. All participants provided written informed consent.

### 2.1. Variables and Measurement

Upon recruitment, women completed a set of questionnaires. Data for the current analysis were extracted from the baseline questionnaire only. This was administered during the first point of contact with the participants recruited between May 2017 and March 2020. The gestational age at recruitment varied between the participants with women being recruited on average at about 6 months (mean ± SD: 5.9 ± 2.4). The questionnaire includes 67 questions collecting data on demographics, psychosocial factors, previous pregnancies, and behaviors during the current pregnancy. The demographic and pregnancy-related characteristics included maternal age, number of children (parity), complications in previous pregnancies including preterm birth, low birth weight, miscarriages, and stillbirth, gestational age, pregnancy planning status, maternal and paternal smoking status, consanguinity, maternal education and employment, number of people living in the house, perceived social support, and childbirth anxiety.

Women were asked about GDM diagnosis in previous pregnancies with the question: “Have you ever had GDM?”, where they could answer “Yes” or “No”. The age at their diagnosis was determined using the question: “If yes, how old were you when you were diagnosed with GDM?”, requesting women to input the age in years. The difference between age at current index pregnancy and age when diagnosed with the first previous GDM was used as an estimation of time since first GDM diagnosis. Women diagnosed with previous GDM were further categorized into two groups according to the time since first diagnosis with GDM (≤5 years since diagnosis, >5 years since diagnosis) and all other women with no history of previous GDM were included in the comparison group. We did not include women with previous type 1 or diabetes mellitus in the analyses.

Data on preconception and prenatal behaviors before and during this current pregnancy were also extracted from the questionnaire. Pregnant women reported whether they planned their pregnancy by answering yes or no to the question: “Was this pregnancy planned?”. Contraceptive use was coded as a yes or no if the women reported the use of any of the methods in the question: “Have you or your husband at any time during the last year used the following methods to avoid becoming pregnant?”. For supplement use, women could report if they used the supplement daily, weekly, monthly, or never to the question: “Do you take any medications or supplements without a doctor’s prescription?”. Women were reported as using the supplement if they answered “Daily” to this question. If they reported using it weekly, monthly or never, they were coded as not using the supplement as folate, vitamin D, multivitamins, and iron are to be consumed daily as per prenatal guidelines. Women were also queried on their physical activity and sedentary behaviors. If the women answered “1–2 times weekly”, “3–5 times weekly”, or “Daily” to the question: “How often are you physically active in your leisure time to the extent that you get out of breath or sweat?”, they were coded as physically active “Ever”, while those who responded “Never” were coded physically active “Never”. With respect to sedentary behavior, women indicated the number of hours they spent sitting during the week and weekend. This was left as a continuous variable truncating the hours to a maximum of 16 hours a day to incorporate sleeping periods (assuming eight hours of sleep). Worrying about the upcoming birth was coded as “Yes” if the women responded “Yes, quite a lot” or “Yes, sometimes” to the question: “Do you worry about the upcoming birth?”. Similarly, they were coded as “No” if the women responded “No, not much” or “No, not at all”. Women were asked to describe their previous birth complications using the questions, “Have any of the following conditions occurred with any of your last pregnancies”. Women could answer “Yes”, “No”, or “I do not know” to any of the four options, which were “Birth weight 2.5 kg or less”, “Baby born three weeks before the full-term pregnancy (before 37 weeks)”, “Miscarriage”, “Stillbirth”.

### 2.2. Statistical Analyses

Descriptive statistics were performed to show and compare the distribution of characteristics of the study population by previous diagnosis of GDM status. Continuous variables were presented as means and standard deviations and categorical variables as counts and percentages. Student t-test was used between group means for continuous variables and Pearson chi-square test was used to determine differences in proportions for categorical variables. Univariate and multivariate regression models were used to quantify the association between history of GDM and various maternal factors and behaviors during the current pregnancy. Crude and adjusted odds ratios (aOR) with 95% confidence intervals (CI) were calculated. Statistical analyses were performed using Stata 16.1 (Stata Corp, College Station, TX, USA). A *p*-value less than or equal to 0.05 and CI were used to determine statistical significance.

## 3. Results

A total of 7899 pregnant women were recruited from May 2017 to March 2020. Out of these, 5912 pregnant women were multiparous, meaning this was not their first pregnancy and, hence, they qualified to respond to the history of previous GDM question. After excluding those with missing responses, 5738 (72.6% of total) pregnant women had valid responses to whether they had been diagnosed with GDM in their past pregnancies and were included in this analysis. Out of 5738 pregnant women included in this study, 1684 (29.4%) reported a previous diagnosis of GDM. Three-quarters (*n* = 1308; 77.7%) of the 1684 women with a history of previous GDM had information on the age they were first diagnosed. More than half (*n* = 684; 52.3%) of the pregnant women with a history of GDM had a recent diagnosis of GDM (≤5 years since first diagnosis), and 47.7% (*n* = 624) had a diagnosis more than 5 years prior to this index pregnancy.

Women with a history of previous GDM were older by approximately 3 years (34.1 versus 31.5 years) at the index pregnancy compared to those with no history of previous GDM. They reported having more children and slightly younger age of menarche (12.8 versus 13.0 years) compared to those with no history of previous GDM. Pregnant women with a history of GDM also showed higher proportions of employment (38.0% versus 33.9%), previous infertility treatment (11.7% versus 8.5%), and not being in a consanguineous marriage (54.5% versus 47.3%). They also reported more events of any previous birth complications (65.1% versus 62.2%), such as low birth weight (40.7% versus 37.0%), premature births (21.5% versus 16.9%), miscarriages (44.9% versus 39.0%), or stillbirth (21.5% versus 16.9%) compared to pregnant women with no history of previous GDM (Table 1).

**Figure 1:**
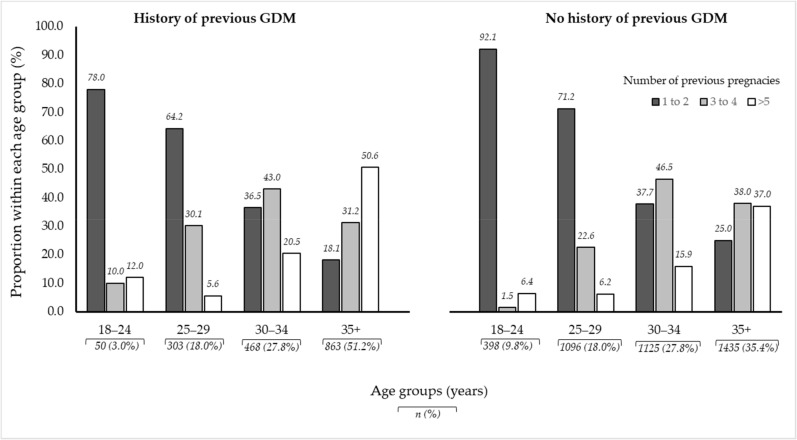
Illustration of age and gravidity proportions between women with a history of previous GDM and those with no history of previous GDM amongst 5738 pregnant women in Al Ain, UAE. The Mutaba’ah Study.

Figure 1 illustrates the distribution of age and gravidity by previous GDM status in the study population. Older women with a history of GDM were more likely to be grand multigravida compared to older women with no history of GDM (50.6% versus 37.0%, *p* < 0.001).

Activities and behaviors before and during the index pregnancy showed only slight differences between pregnant women with and without history of previous GDM. Women who experienced previous GDM were less likely to have a planned pregnancy for the index pregnancy (50.2% versus 55.7%, *p* < 0.0001). Other behaviors such as supplement use (both in composite variables and in singular supplement use), physical activity before and during pregnancy, sedentary behavior during the weekdays and weekends, or childbirth anxiety during pregnancy showed no significant differences by history of previous GDM status (Table 2).

The crude regression analysis showed that women with a history of previous GDM were less likely to have planned pregnancies (OR: 0.80, 95% CI 0.71–0.90) compared to pregnant women with no history of GDM (Table 3). After adjusting for potential confounders including age, parity, employment, age of menarche, and infertility treatment, pregnant women with a previous GDM diagnosis retained the significant lower odds of having planned pregnancies (aOR: 0.84, 95% CI 0.74–0.96). They were also more likely to have had shorter interpregnancy intervals (aOR: 0.88, 95% CI: 0.82–0.94) and be worried about giving birth (aOR 1.18, 95% CI: 1.03–1.36) compared to women with no history of GDM. No significant differences were detected between pregnant women with and without history of previous GDM in levels of physical activity, sedentary behavior, or supplement use before or during this current pregnancy (Table 3).

**Figure 2:**
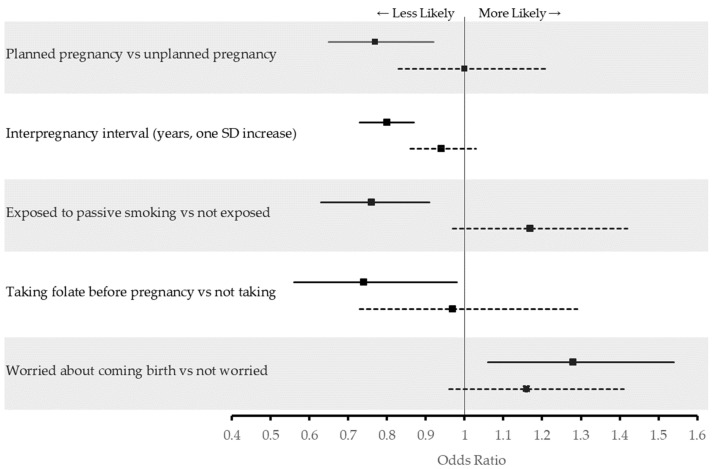
Adjusted odds ratios of activities and behaviors during^*^ current pregnancy associated with time since first diagnosis of previous GDM amongst 5738 pregnant women in Al Ain, UAE. The Mutaba’ah Study. Solid lines indicate adjusted odds ratios (aOR) and 95% confidence interval (CI) among women with a history of previous GDM who had ≤5 years since their first diagnosis while dotted lines indicate aOR and 95% CI among women with a history of previous GDM who had >5 years since their first diagnosis compared to women with no history of previous GDM. ^*^ Folate consumption before current pregnancy.

Figure 2 shows the adjusted odds ratios of activities and behaviors during the current pregnancy associated with time since first diagnosis of previous GDM. Women with a shorter time (≤5 years) between their first GDM diagnosis and current pregnancy were less likely to have planned their index pregnancy (aOR: 0.77, 95% CI 0.65–0.92), had shorter interpregnancy intervals (aOR: 0.80, 95% CI 0.73–0.87, per SD increase), were less likely to supplement with folate before their index pregnancy (aOR: 0.74, 95% CI: 0.56–0.98), and were less likely to be exposed to passive smoking (aOR: 0.76, 95% CI 0.63–0.91). However, they were more likely to worry about their upcoming birth (aOR: 1.28, 95% CI 1.06–1.54). All associations between the three categories of previous diagnosis can be found in the Supplementary Materials (Table S1).

## 4. Discussion

This study provides the first estimates on the associations between GDM history and pre-pregnancy and prenatal behaviors during a subsequent pregnancy. Nearly a third (29.4%) of parous pregnant women in the study population reported a history of GDM in a previous pregnancy, and approximately 48% of them had the first GDM diagnosis more than 5 years before this current pregnancy. There were no significant differences between women with and without a history of GDM in supplement use, sedentary behavior, or physical activity before and during this current pregnancy.

Supplements such as folic acid and vitamin D have been shown to be associated with better pregnancy outcomes [18,27]. Overall, only a third of all pregnant women in this study reported taking specific prenatal supplements (e.g., folic acid, iron, and vitamin D) on a daily basis. Although not significant, the use of any supplement before and during this current pregnancy was even less in pregnant women with history of GDM than in those without a history of GDM. Nevertheless, in the stratified analysis, there was a significant lack of folic acid supplementation among women with a history of recent diagnosis of GDM. This reinstates the need for educating women of childbearing age with a history of GDM on the importance of appropriate supplementation with the guidance of their physician. On the other hand, less than half of all pregnant women in this study reported participating in physical activity at least once a week. This finding has clear public health importance as physical activity can assist with weight management and improve insulin sensitivity, both of which reduce the risk of GDM [17].

The study findings showed that women with a history of previous GDM were less likely to plan their pregnancies. This seems to be driven by the subset of women who were recently diagnosed (≤5 years). To our knowledge, this association has not been studied in other populations. With recurrence rates of GDM as high as 73% [23], it is necessary to further research the potential negative consequences of unplanned pregnancies in women with history of previous GDM. Planned pregnancies may entail that women adopt better behaviors during the preconception and early prenatal period [28] to maximize the likelihood of a healthy full-term pregnancy. Conversely, unplanned pregnancies may lead the woman to not seek care earlier during their pregnancy (i.e., late antenatal care initiation) or be prepared to manage a difficult pregnancy [29,30]. In this study population, previous research reported that half of the pregnant women were late in initiating their ANC (>4 months’ gestation) [30], which may lead to the late identification of pregnancy complications, such as GDM. Appropriate ANC initiation is especially important amongst pregnant women with a history of GDM and targeted public health campaigns should encourage all women to initiate ANC within the first four months of pregnancy regardless of maternal age, parity, or previous complications such as GDM. Women with a shorter period between their first previous GDM diagnosis and current pregnancy were more likely to have less spacing between their children. This could be an indication of an unplanned pregnancy as shorter interpregnancy intervals are usually linked to unintended pregnancies [31]. Women with previous GDM may also have been worried about the consequences of GDM on their reproductive life and hence, decided to have a shorter interpregnancy interval. There was no difference between women with and without a history of GDM in contraception uptake suggesting that women with a history of GDM might benefit from further education. This could include contraceptive use and family planning, in addition to the relationship between optimal pregnancy intervals and unplanned pregnancies and healthy pregnancy outcomes [32]. Nonetheless, on average, this study population was spacing their children approximately 33 months apart, which is in line with the World Health Organization guidelines of appropriate interpregnancy interval - suggesting that a minimum of 18 months should be kept between children [33]. Interestingly, there was a significant negative association between a previous GDM diagnosis closer to the current pregnancy and prenatal exposure to passive smoking. Recent research has shown that exposure to environmental tobacco smoke during pregnancy is associated with a range of negative maternal and infant health outcomes. Passive smoking can lead to intrauterine growth retardation, stillbirth, congenital abnormalities, and further issues in childhood such as sudden infant death syndrome and asthma [34,35]. Pregnant women with previous GDM reported more events of any previous birth complications, such as low birth weight, premature births, miscarriages, or stillbirth compared to those with no history of previous GDM. The proportion of previous birth complications, although relatively high, is a cumulative percentage combining all birth complications in previous reproductive life. Such high rates of complications throughout the reproductive career has been shown in literature [36,37,38,39]. However, this study could not determine the associations between history of GDM and birth complications due to the lack of temporal information of these events during the previous pregnancies.

To our knowledge, this study is the first to explore the relationship between a history of previous GDM and health behaviors during and before the current pregnancy. This analysis did not investigate whether the overall prenatal behaviors in this population of pregnant women, including those with no history of previous GDM, were optimal or not. The findings of this study show that women with a previous history of GDM were not too different than their counterparts with no history of GDM in the adoption of better GDM-related behaviors such as increasing physical activity, reducing sedentary behavior, and appropriate supplement use. One plausible explanation for this observation may be that the adoption of healthy lifestyles by women with previous GDM were only for short periods, i.e., during the pregnancy when they were diagnosed with GDM or before and during one subsequent pregnancy but not beyond that. This could not be explored here as the analysis did not include information on whether the previous GDM was immediately before this current pregnancy. However, it indicates that lifestyle counseling may facilitate and empower women with a history of GDM for the long-term adoption of healthy lifestyle behaviors. This may be so even after a successful GDM-free pregnancy to ensure that they are in optimal health before any future pregnancy. Advice on lifestyle behaviors such as physical activity, energy-balanced and nutrient-dense diets, weight management, and appropriate supplementation may come from an array of interventions. Compared to traditional ANC, electronic-health (e-health) provision has shown to be feasible and lead to increased perceived confidence of dietary change and readiness to exercise. This suggests that e-health might be effective at empowering women to adopt healthier lifestyles [40]. Moreover, social media interventions have been shown to improve health literacy and may be a preferable alternative to traditional face-to-face lifestyle counseling. This may be appealing to women who might feel stigmatized for having certain lifestyle behaviors or diseases during pregnancy [41,42].

A major strength of the study is the large representative sample of pregnant women with detailed information on a wide range of health behaviors. However, this analysis was based on self-reported data, and the answers to the questions were not confirmed from the medical records. Although, there is minimal chance of recall bias of previous adverse outcomes such as GDM, recalling the age at diagnosis of first previous GDM and activities and behaviors before the current pregnancy might introduce possibilities of recall bias. Moreover, there may have been other factors that the study did not collect data on, and therefore, could not have been adjusted for in the analysis leading to potential residual confounding. Nonetheless, the longitudinal data of the Mutaba’ah Study over subsequent pregnancies will allow for future estimates of the risk of recurrent GDM and the associated effects on maternal and infant health outcomes. This will allow for identifying modifiable risk factors that can be used in targeted preconception and prenatal interventions.

## 5. Conclusions

Nearly a third of pregnant women in this study population had a history of GDM in a previous pregnancy. Pregnant women with a previous history of GDM were no different than their counterparts with no history of GDM in the adoption of healthy lifestyle behaviors that decrease the risk of recurring of GDM during this current pregnancy. They were less likely to plan their index pregnancies and consume folate before this current pregnancy. More intensive and long-term lifestyle counseling, possibly supported by e-health and social media materials, might be required to empower pregnant women with history of GDM to adopt and maintain healthy lifestyle behaviors that decrease the risk of GDM recurrence and the consequential adverse maternal and infant health outcomes.

## Figures and Tables

**Table 1:** Characteristics of 5738 pregnant women by history of previous gestational diabetes mellitus (GDM) status in Al Ain, UAE. The Mutaba’ah Study.

	No Previous History of GDM	Previous History of GDM	Time Since First Diagnosis of Previous GDM	
			≤5 Years	>5 Years
*N*	4054 (70.6%)	1684 (29.4%)	684 (52.3%)	624 (47.7%)
Age (years) *	31.4 ± 0.89	34.0 ± 0.14	32.2 ± 0.20	36.5 ± 0.18
Number of children *	2.8 ± 0.03	3.6 ± 0.05	2.8 ± 0.74	4.3 ± 0.08
Age of menarche (years) *	13.0 ± 0.03	12.8 ± 0.05	13.2 ± 0.08	12.7 ± 0.07
Employment *				
Yes	1338 (33.9%)	629 (38.0%)	259 (38.4%)	243 (39.5%)
No	2610 (66.1%)	1025 (62.0%)	415 (61.6%)	373 (60.6%)
Education *				
High school and below	2373 (60.0%)	1002 (60.5%)	396 (58.7%)	377 (61.3%)
More than high school	1582 (40.0%)	653 (39.5%)	278 (41.3%)	238 (38.7%)
Consanguinity *				
Yes	968 (52.7%)	360 (45.5%)	129 (48.9%)	126 (46.7%)
No	867 (47.3%)	431 (54.5%)	135 (51.1%)	144 (53.3%)
Infertility treatment *				
Yes	338 (8.5%)	192 (11.7%)	81 (12.0%)	82 (13.3%)
No	3636 (91.6%)	1453 (88.3%)	594 (88.0%)	533 (86.7%)
Any previous birth complications *				
Yes	2522 (62.2%)	1096 (65.1%)	409 (59.8%)	425 (68.1%)
No	1532 (37.8%)	588 (34.9%)	275 (40.2%)	199 (31.9%)

* Indicates *p* values less than 0.05 for comparison between no previous history of GDM and previous history of GDM.

**Table 2:** Distributions of activities and behaviours before and during current pregnancy by history of previous GDM status amongst 5738 pregnant women in Al Ain, UAE. The Mutaba’ah Study.

	No Previous History of GDM	Previous History of GDM	Time Since First Diagnosis of Previous GDM	
			≤5 Years	>5 Years
**Activities and behaviors before current pregnancy**				
Planned pregnancy *				
Yes	2227 (55.7%)	831 (50.1%)	344 (50.6%)	324 (52.3%)
No	1770 (44.2%)	826 (49.9%)	336 (49.4%)	296 (47.7%)
Contraceptive use				
Yes	1238 (56.7%)	492 (54.2%)	165 (57.9%)	188 (60.8%)
No	944 (43.3%)	415 (45.8%)	120 (42.1%)	121 (39.2%)
Interpregnancy interval	34.2 ± 0.64	33.0 ± 0.90	27.1 ± 1.4	39.0 ± 1.2
Physical activity				
Ever	1610 (44.0%)	677 (44.1%)	282 (44.3%)	275 (47.3%)
Never	2050 (56.0%)	857 (55.9%)	355 (55.7%)	306 (52.7%)
Any supplement use				
Yes	831 (26.6%)	316 (24.8%)	118 (23.3%)	118 (25.1%)
No	2290 (73.4%)	958 (75.2%)	389 (76.7%)	352 (74.9%)
Folic acid				
Yes	541 (18.0%)	205 (16.6%)	68 (13.9%)	85 (18.6%)
No	2467 (82.0%)	1028 (83.3%)	421 (86.1%)	372 (81.4%)
Iron consumption				
Yes	298 (10.1%)	102 (8.4%)	42 (8.8%)	31 (6.9%)
No	2648 (89.9%)	1107 (91.6%)	436 (91.2%)	417 (93.1%)
Vitamin D				
Yes	183 (9.6%)	71 (9.1%)	27 (9.9%)	19 (7.1%)
No	1715 (90.4%)	712 (90.9%)	246 (90.1%)	246 (92.8%)
Multivitamin consumption				
Yes	330 (11.2%)	114 (9.4%)	47 (9.7%)	41 (9.3%)
No	2606 (88.8%)	1101 (90.6%)	440 (90.3%)	400 (90.7%)
**Activities and behaviors during current pregnancy**				
Worried about upcoming birth				
Yes	2532 (64.8%)	1085 (66.6%)	458 (69.1%)	393 (65.0%)
No	1373 (36.2%)	545 (33.4%)	205 (30.9%)	212 (35.0%)
Physical activity				
Ever	1684 (45.4%)	717 (46.4%)	315 (49.2%)	254 (43.6%)
Never	2024 (54.6%)	828 (53.6%)	325 (50.8%)	328 (56.4%)
Sedentary behavior overall (hours)	6.3 ± 0.06	6.2 ± 0.09	6.3 ± 0.13	6.1 ± 0.14
Sedentary behavior during week (hours)	6.2 ± 0.06	6.2 ± 0.09	6.3 ± 0.15	6.1 ± 0.15
Sedentary behavior during weekend (hours)	6.5 ± 0.06	6.3 ± 0.10	6.2 ± 0.15	6.1 ± 0.15
Smoking				
Yes	31 (0.7%)	7 (0.4%)	2 (0.3%)	3 (0.5%)
No	3986 (99.2%)	1657 (99.6%)	675 (99.7%)	614 (99.5%)
Exposure to passive smoking				
Yes	1382 (34.3%)	598 (35.7%)	196 (28.8%)	211 (34.0%)
No	2646 (65.7%)	1075 (64.3%)	485 (71.2%)	409 (66.0%)
Any supplement use				
Yes	1192 (39.1%)	454 (36.3%)	184 (36.3%)	155 (33.7%)
No	1860 (60.9%)	798 (63.7%)	323 (63.7%)	305 (66.3%)
Folic acid				
Yes	959 (31.3%)	353 (28.3%)	148 (29.2%)	123 (26.8%)
No	2101 (68.7%)	896 (71.7%)	359 (70.8%)	336 (73.2%)
Iron consumption				
Yes	654 (21.9%)	260 (21.2%)	120 (24.1%)	78 (17.5%)
No	2330 (78.1%)	967 (78.8%)	377 (75.9%)	369 (82.5%)
Vitamin D				
Yes	526 (17.7%)	207 (17.1%)	93 (18.8%)	61 (13.8%)
No	2450 (82.3%)	1007 (83.9%)	402 (81.2%)	381 (86.2%)
Multivitamin consumption				
Yes	640 (21.5%)	245 (20.1%)	108 (21.7%)	80 (18.1%)
No	2330 (78.5%)	975 (79.9%)	390 (78.3%)	363 (81.9%)

* Indicates significant differences between the groups.

**Table 3:** Associations between history of previous GDM and activities and behaviors before and during the current pregnancy amongst 5738 pregnant women in Al Ain, UAE. The Mutaba’ah Study.

	Crude Odds Ratio	Adjusted Odds Ratio ^a^
**Activities and behaviors before current pregnancy**		
**Planned pregnancy**	**0.80 (0.71–0.90)**	**0.84 (0.74–0.96)**
Contraceptive use	0.92 (0.79–1.07)	0.89 (0.74–1.08)
**Interpregnancy interval (SD)**	**0.96 (0.96–1.02)**	**0.88 (0.82–0.94)**
Physical activity	1.00 (0.88–1.13)	0.96 (0.84–1.10)
Any supplement use	0.91 (0.78–1.06)	0.94 (0.79–1.12)
Folic acid consumption	0.93 (0.78–1.10)	0.91 (0.74–1.12)
Iron consumption	0.83 (0.66–1.05)	0.93 (0.71–1.23)
Vitamin D consumption	0.97 (0.73–1.28)	1.06 (0.75–1.50)
Multi-vitamin consumption	0.82 (0.65–1.01)	0.80 (0.61–1.03)
**Activities and behaviors during current pregnancy**		
**Worried about birth**	**1.09 (0.97–1.23)**	**1.18 (1.03–1.36)**
Physical activity	1.04 (0.93–1.17)	1.05 (0.92–1.20)
Sedentary behavior overall	0.89 (0.73–1.09)	0.90 (0.72–1.13)
Sedentary behavior during week	0.95 (0.76–1.17)	0.95 (0.74–1.21)
Sedentary behavior during weekend	0.78 (0.62–0.98)	0.81 (0.62–1.04)
Smoking	0.61 (0.28–1.32)	0.58 (0.21–1.59)
Exposure to passive smoking	1.06 (0.94–1.19)	1.11 (0.96–1.27)
Any supplement use	0.94 (0.83–1.07)	1.00 (0.85–1.17)
Folic acid consumption	0.89 (0.77–1.02)	0.95 (0.80–1.12)
Iron consumption	0.97 (0.83–1.14)	1.03 (0.85–1.24)
Vitamin D consumption	0.97 (0.82–1.16)	1.09 (0.89–1.33)
Multi-vitamin consumption	0.93 (0.79–1.09)	0.96 (0.80–1.17)

^a^ Adjusted for age, parity, infertility treatment, age of menarche, and employment. Bolded variable indicates significant associations.

## Data Availability

The data presented in this study are available on request from the Mutaba’ah Study.

## Supplementary Materials

The following is available online at https://www.mdpi.com/1660-4601/18/1/58/s1, Table S1: Crude and adjusted odds ratio stratified by time since first diagnosis of gestational diabetes mellitus with reference to women with no history previous of GDM amongst 5738 pregnant women in Al Ain, UAE. The Mutaba’ah Study.
