# Supplementary material for: Effect of Gestational Diabetes Mellitus History on Future Pregnancy Behaviors: The Mutaba’ah Study
Source: Int J Environ Res Public Health. 2020 Dec 23;18(1):58. doi: 10.3390/ijerph18010058 (PMC7796159; doi:10.3390/ijerph18010058)
Supplement: Supplementary file 1 [file ijerph-18-00058-s001.pdf]

Table S1: Crude and adjusted odds ratio stratified by time since first diagnosis of gestational diabetes mellitus with reference to women with no history previous of GDM amongst 5,738 pregnant women in Al Ain, UAE. The Mutaba'ah Study

	Crude OR with 95% CI	Adjusted OR with 95% CI
**Activities and behaviors before current pregnancy**		
**Planned pregnancy**		
No previous GDM	1	1
≤ 5 years since first diagnosis	0.81 (0.69-0.96)	0.77 (0.65-0.92)
> 5 years since first diagnosis	0.87 (0.73-1.03)	1.00 (0.83-1.21)
**Contraceptive use**		
No previous GDM	1	1
≤ 5 years since first diagnosis	1.04 (0.82-1.34)	1.08 (0.83-1.41)
> 5 years since first diagnosis	1.18 (0.93-1.51)	0.91 (0.69-1.19)
**Inter Pregnancy Interval (SD)**		
No previous GDM	1	1
≤ 5 years since first diagnosis	0.82 (0.77-0.90)	0.80 (0.73-0.87)
> 5 years since first diagnosis	1.14 (1.04-1.24)	0.94 (0.86-1.03)
**Physical Activity**		
No previous GDM	1	1
≤ 5 years since first diagnosis	1.01 (0.85-1.20)	0.99 (0.83-1.19)
> 5 years since first diagnosis	1.15 (0.96-1.36)	1.09 (0.90-1.32)
**Folic acid consumption**		
No previous GDM	1	1
≤ 5 years since first diagnosis	0.74 (0.56-0.97)	0.74 (0.56-0.98)
> 5 years since first diagnosis	1.04 (0.81-1.34)	0.97 (0.73-1.29)
**Iron consumption**		
No previous GDM	1	1
≤ 5 years since first diagnosis	0.86 (0.61-1.20)	0.95 (0.67-1.35)
> 5 years since first diagnosis	0.66 (0.45-0.97)	0.64 (0.42-1.00)
**Vitamin D consumption**		
No previous GDM	1	1
≤ 5 years since first diagnosis	1.03 (0.68-1.58)	1.11 (0.71-1.73)
> 5 years since first diagnosis	0.73 (0.45-1.19)	0.87 (0.50-1.50)
**Multi-vitamin consumption**		
No previous GDM	1	1
≤ 5 years since first diagnosis	0.84 (0.61-1.16)	0.84 (0.60-1.17)
> 5 years since first diagnosis	0.81 (0.58-1.14)	0.78 (0.54-1.13)
**Activities and behaviors during current pregnancy**		
**Worried about birth**		
No previous GDM	1	1
≤ 5 years since first diagnosis	1.21 (1.01-1.44)	1.28 (1.06-1.54)
> 5 years since first diagnosis	1.00 (0.84-1.20)	1.16 (0.96-1.41)

**Physical Activity**		
No previous GDM	1	1
≤ 5 years since first diagnosis	1.17 (0.99-1.38)	1.16 (0.97-1.38)
> 5 years since first diagnosis	0.93 (0.78-1.11)	0.99 (0.82-1.20)
**Sedentary behavior overall**		
No previous GDM	1	1
≤ 5 years since first diagnosis	0.94 (0.70-1.26)	0.87 (0.65-1.17)
> 5 years since first diagnosis	0.79 (0.58-1.06)	0.88 (0.64-1.21)
**Sedentary behavior during week**		
No previous GDM	1	1
≤ 5 years since first diagnosis	1.01 (0.74-1.38)	0.94 (0.69-1.29)
> 5 years since first diagnosis	0.84 (0.61-1.16)	0.92 (0.66-1.30)
**Sedentary during weekend**		
No previous GDM	1	1
≤ 5 years since first diagnosis	0.77 (0.56-1.07)	0.73 (0.52-1.02)
> 5 years since first diagnosis	0.66 (0.47-0.92)	0.76 (0.53-1.10)
**Exposure to passive smoking**		
No previous GDM	1	1
≤ 5 years since first diagnosis	0.77 (0.65-0.92)	0.76 (0.63-0.91)
> 5 years since first diagnosis	0.99 (0.83-1.18)	1.17 (0.97-1.42)
**Folic acid consumption**		
No previous GDM	1	1
≤ 5 years since first diagnosis	0.90 (0.73-1.11)	0.93 (0.75-1.16)
> 5 years since first diagnosis	0.80 (0.64-0.99)	0.92 (0.73-1.18)
**Iron consumption**		
No previous GDM	1	1
≤ 5 years since first diagnosis	1.13 (0.91-1.42)	1.20 (0.95-1.51)
> 5 years since first diagnosis	0.75 (0.58-0.98)	0.89 (0.67-1.18)
**Vitamin D consumption**		
No previous GDM	1	1
≤ 5 years since first diagnosis	1.08 (0.84-1.38)	1.16 (0.90-1.50)
> 5 years since first diagnosis	0.75 (0.56-0.99)	0.90 (0.66-1.23)
**Multi-vitamin consumption**		
No previous GDM	1	1
≤ 5 years since first diagnosis	1.01 (0.80-1.27)	1.03 (0.81-1.31)
> 5 years since first diagnosis	0.80 (0.62-1.04)	0.90 (0.68-1.19)
